# Contributions of Imaging to Neuromodulatory Treatment of Drug-Refractory Epilepsy

**DOI:** 10.3390/brainsci10100700

**Published:** 2020-10-02

**Authors:** Niels Alexander Foit, Andrea Bernasconi, Neda Ladbon-Bernasconi

**Affiliations:** 1 Neuroimaging of Epilepsy Laboratory, McConnell Brain Imaging Centre, Montreal Neurological Institute and Hospital, McGill University, Montreal, QC H3A2B4, Canada; andrea.bernasconi@mcgill.ca (A.B.); neda.ladbon-bernasconi@mcgill.ca (N.L.-B.); 2 Department of Neurosurgery, Medical Center–University of Freiburg, Faculty of Medicine, D-79106 Freiburg, Germany

**Keywords:** epilepsy, imaging, MRI, networks, neuromodulation

## Abstract

Epilepsy affects about 1% of the world’s population, and up to 30% of all patients will ultimately not achieve freedom from seizures with anticonvulsive medication alone. While surgical resection of a magnetic resonance imaging (MRI) -identifiable lesion remains the first-line treatment option for drug-refractory epilepsy, surgery cannot be offered to all. Neuromodulatory therapy targeting “seizures” instead of “epilepsy” has emerged as a valuable treatment option for these patients, including invasive procedures such as deep brain stimulation (DBS), responsive neurostimulation (RNS) and peripheral approaches such as vagus nerve stimulation (VNS). The purpose of this review is to provide in-depth information on current concepts and evidence on network-level aspects of drug-refractory epilepsy. We reviewed the current evidence gained from studies utilizing advanced imaging methodology, with a specific focus on their contributions to neuromodulatory therapy.

## 1. Introduction

Epilepsy affects about up to 1% of the world’s population [1], and 1/3 of all patients will not be rendered seizure-free by anticonvulsive medication [2]. The two most common medically refractory epilepsy syndromes include temporal lobe epilepsy (TLE) with hippocampal sclerosis as its pathological hallmark [3] and extra-temporal lobe epilepsy arising developmental malformations, particularly focal cortical dysplasia (FCD) [4]. Surgical resection of an MRI-identifiable lesion is the first-line treatment option for drug-refractory epilepsy [5] with success rates that may reach up to 80% [6]. Notably, complete resection of the lesion is the most reliable predictor for postoperative seizure freedom [7].

Despite its efficacy, resective surgery cannot be offered to all patients with refractory seizures. Specifically, bilateral or multifocal seizures, proximity to or overlap of the ictal zone with eloquent cortical areas, and lack of distinctive imaging abnormalities represent potential surgical contraindications [8]. Moreover, depending on the resected brain area, patients may develop a new neurologic or cognitive deficit postoperatively or worsen an existing one [9]. From a network level perspective, evidence from preclinical models and human studies indicate that specific cortical and subcortical networks are key elements in the genesis and expression of seizures [10], supporting the hypothesis that pathological substrates of refractory epilepsy may be less “focal” than traditionally presumed [11].

Neuromodulation refers to the process of electrically or chemically altering signal transmission of the central nervous system through implantable devices to excite, inhibit or modify neuronal activity, to elicit therapeutic effects [12]. It is a rapidly evolving field on the boundary between biomedical and engineering research across a wide range of scientific disciplines [12]. In medicine, neuromodulatory therapy aims at improving quality of life by means of neurostimulators or implantable drug-delivery systems [13]. Implantable devices are currently available for a variety of conditions, including movement disorders, chronic pain or psychiatric disorders. Neuromodulatory therapy targeting “seizures” instead of “epilepsy” [14] has recently emerged as a valuable treatment option for non-operable patients, including invasive procedures such as deep brain stimulation (DBS), responsive neurostimulation (RNS), peripheral approaches such as vagus nerve stimulation (VNS) or non-invasive, transcranial approaches. The purpose of this review is to provide in-depth information on current concepts and evidence on drug-refractory epilepsy as a network disorder. We reviewed the current state of evidence gained from advanced neuroimaging studies, with a specific focus on their potential contribution to neuromodulatory therapy.

## 2. Contributions of Advanced Neuroimaging to Presurgical Evaluation of Drug-Resistant Epilepsy

### 2.1. Network Modelling Using Structural and Functional MRI

From a basic perspective, a network is formed by a given number of items that exhibit pairwise associations [15]. The brain as a whole is a hierarchically organized network [16], partitioned into densely connected units spanning from local circuits to broad functional areas [17]. Continuing methodological advancements in neuroimaging now allow for non-invasive investigations of both structural and functional networks in vivo. Information on structural networks [16] is derived from diffusion MRI tractography or covariance of morphological markers, e.g., gray matter volume or cortical thickness [18], representing physical connections. Diffusion imaging provides voxel-wise information on magnitude and directionality of water diffusion and is utilized to assess white matter connective circuitry [19]. Moreover, the use of tractography algorithms allows for a reconstruction of fiber pathways along plausible diffusion trajectories, which have been cross-validated against anatomical tract-tracing studies [20]. Structural covariance analysis may be utilized to assess changes of anatomical connectivity between cortical areas [21]. This methodology infers networks from morphological markers, i.e., gray matter volume or cortical thickness. A probable interconnection between given regions is derived from high inter-correlation of morphological markers [22]. Covariance patterns may reflect trophic and/or signaling interactions between brain areas and exhibit close overlap with networks derived from diffusion imaging or resting-state functional MRI [23].

Functional connectivity is usually estimated from statistical relationships of neurophysiological signals between brain regions [24], with time-series extracted from sources such as task-based or resting-state functional MRI [25]. These sequences are nowadays included in presurgical evaluation protocols, mainly to localize eloquent areas, e.g., hemispherical language dominance. Resting-state functional MRI offers several advantages over task-based paradigms, e.g., high reproducibility among subjects [26]. However, compared to task-based measures, its yield to infer function has not yet been solidly established, hindering introduction into routine clinical use.

### 2.2. Studying Networks with Graph Theory

**Figure 1:**
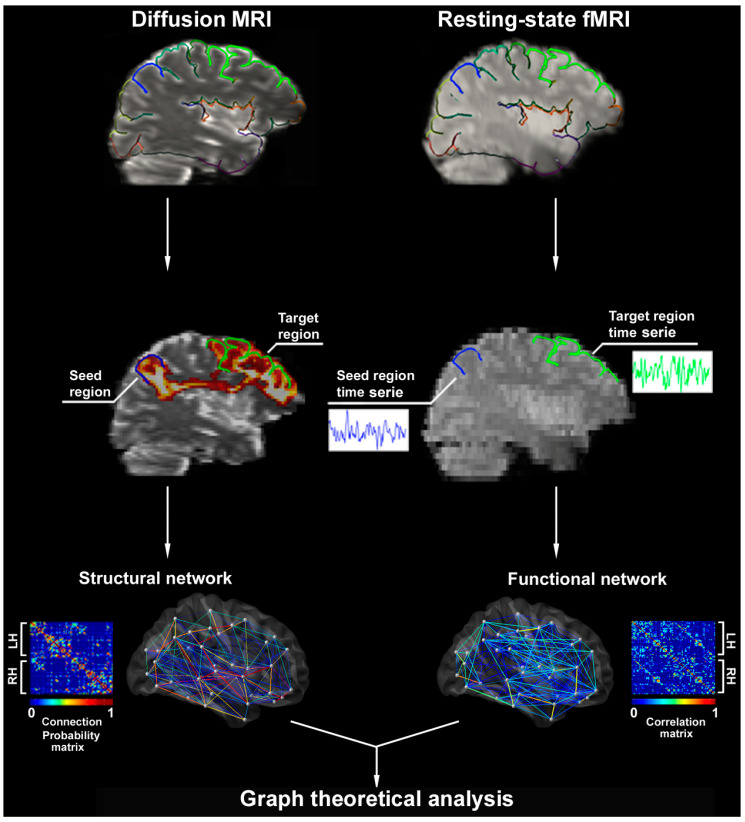
Construction of structural and functional networks. Upper panels show parcellated surfaces (color-coded by lobes) mapped onto the diffusion and resting-state fMRI. In the middle panels, each parcel represents a seed and its connectivity is estimated to all other parcels. Diffusion tensor-probabilistic tractography and functional MRI time series correlations between the superior frontal (**green**) and the post-central (**blue**) parcels are shown. Lower panels display connection probabilities (represented by partial correlation coefficients between all pairs of parcels) used to generate structural and functional association matrices. Matrices are the substrate for graph-theoretic analyses of network properties. In the graph, nodes represent parcels (white dots) and edges (lines linking nodes) pairwise connections color-coded according to connectivity strength. *Abbrev*: LH/RH: left/right hemisphere.

Graph theory offers a powerful framework for the mathematical representation and analysis of complex systems and allows for the quantification of organizational patterns of brain networks [15]. In graph-theory terms, a network contains a collection of *nodes* (brain regions) connected by *edges* (structural or functional connections; Figure 1) [15]. A multitude of criteria can be employed to define nodes, from single voxels to entire brain regions or functional parcellations [27]. By assessing all pairwise connections between a given numbers of nodes, a connectivity matrix can be inferred [28]. Based on shared similarities, groups of nodes can be arranged in clusters (*modules*), demonstrating dense internal connectivity, but relative segregation from the rest of the network. By analyzing metrics of centrality, nodes with high degrees of connectivity and prominent roles can be identified, which are thus referred to as *hubs*. Measures of local efficiency, such as *clustering coefficient,* reflect connectional density within the local environment, whereas *path length* describes the average number of connections between nodal pairs assessing efficiency of information flow at a global scale [29]. These metrics of whole-brain topology have shown *small world* characteristics of brain networks in healthy individuals exhibiting short paths and high clustering [30], a configuration allowing for an efficient information flow.

Another relevant parameter, particularly in epilepsy, stems from the relative importance of a node within a specific network, i.e., centrality [17]. Measures of centrality are useful for charting the global topography of brain networks. Specifically, networks exhibiting higher degrees of interconnections among highly central nodes than expected by chance are considered to represent a rich-club organization [31]. Indeed, rich clubs have been identified in structural connectivity data including the human connectome [32] and reduced rich club connectivity has recently been linked to long-term injury effects of generalized seizures [33]. Rich club connectivity is also altered in epilepsies related to cortical malformations [34,35].

Controllability may be another metric of interest in epilepsy, as it measures the ability to exert control, i.e., induce transition from an initial state to a desired final state. Sparse inhomogeneous networks, which are often found in real-world complex systems, have proven to be most difficult to control, whereas dense and homogeneous networks can be controlled using a few driver nodes [36].

Finally, it should be noted that, irrespective of the investigated modality, a functional core of any human brain network is likely formed by associations between high-centrality nodes of an underlying structural network, thus displaying high overlap between these conceptually separate domains [37].

### 2.3. Epilepsy as a Network Disorder

Clinical manifestations of seizures likely involve similar large-scale brain networks active during the inter-ictal state [38]. A network perspective [39] is therefore of particular relevance, since structures within epileptogenic networks are thought to be involved in both generation and expression of seizures as well as maintenance of the disorder [40]. In this context, non-invasive neuroimaging techniques offer unique opportunities to study networks at multiple levels [25].

Temporal lobe epilepsy (TLE) is the most commonly studied syndrome from a network-level perspective. Recent studies have revealed widespread structural [41] and functional [21] alterations affecting both local circuits and networks at large. Moreover, growing evidence supports the concept that large-scale connectional reconfiguration occurs in epilepsy secondary to cortical malformations [35]. Taken together, these findings prompted a major conceptual shift in the conceptualization of focal epilepsy, emphasizing the importance of a network approach to comprehensively capture the complexity of the disorder. In TLE, graph theoretical analyses have found increased path length, sometimes associated with increased clustering [42]. These changes likely represent pathologically increased local and reduced global network efficiency [11], with similar findings stemming from analyses of connectivity in extra-temporal lobe epilepsy [34].

While a structural brain lesion is considered the core of the epileptogenic focus [43], extra-lesional structural alterations may negatively impact seizure outcome after surgery [44]. Furthermore, distant alterations in morphology [45] and structural connectivity [11] could impair the organization of functional networks [10], promoting both insufficient seizure control [46] and unfavorable cognitive outcomes [47]. It has been demonstrated that TLE patients with favorable seizure outcomes mainly exhibit alterations limited to the resected or disconnected mesial temporal lobe [48]. While the contribution of altered connectivity to seizure outcomes is increasingly recognized [46], single-patient level predictive biomarkers are not yet readily available [44].

Patients who require neuromodulatory treatment for unfavorable seizure control likely exhibit a distributed seizure network with rapid propagation; seizures may be bilateral or even multifocal [49]. Longer epilepsy duration is known to be associated with less favorable surgical outcome, suggesting a progressive disease course with worsening of the structural damage and likely remodeling of seizure-generating networks [50]. Indeed, recent neuroimaging studies found reduced network controllability in drug-refractory TLE [51], which is expected to be even more marked in multifocal epilepsy syndromes. Clearly, understanding the complex interactions between an ictogenic lesion and large-scale brain networks is critical for clinical decision-making [52] and should be investigated in future prospective analyses.

## 3. Imaging-Informed Neuromodulation of Drug-Resistant Epilepsy

### 3.1. Deep Brain Stimulation

The therapeutic principle of DBS is the direct modulation of pathological activity within certain brain networks [53], thus making this approach particularly well suited for network manipulation in drug-refractory epilepsy. DBS involves delivery of predetermined electrical stimulation (open-loop) to given brain structures via stereotactically implanted depth electrodes connected to a pulse generator [8]. While the exact physiological mechanisms still remain poorly understood, DBS is generally considered to exert either inhibitory or excitatory effects, or a combination of both, on target neurons [54,55]. High-frequency stimulation may activate GABA-ergic inhibitory neurons and desynchronize neuronal activity. Low-frequency stimulation potentially reduces overall excitability by induction of long-term depression [56]. More recently, it has been proposed that DBS could disrupt abnormal flow of information in pathological conditions, which seems particularly compelling in the context of epilepsy [54].

Various structures have been targeted by DBS for the treatment of refractory epilepsy in the recent past; however, robust evidence from randomized controlled trials currently only exists for two targets, i.e., the anterior nucleus of the thalamus (ANT) [57] and hippocampus (HC), whereas results for other brain areas such as the cerebellum remain inconclusive [58].

Evidence from the landmark Stimulation of the Anterior Nucleus of the Thalamus in Epilepsy (SANTE) trial [57] revealed a significant decrease in seizure frequency both short and long term and an improved overall quality of life [59], producing Class I-level evidence for the approval of ANT-DBS as a treatment option for refractory epilepsy in the US, Canada, Europe and Australia. Subgroup analyses further demonstrated variable treatment efficacy, with temporal lobe seizures being most responsive, with up to 76% reduction of total seizure frequency [59]. Additionally, there is growing evidence that ANT-DBS may lead to a significant improvement in executive functioning, memory, attention and mood [60]. However, the exact underlying mechanisms of the observed cognitive improvements remain poorly understood and warrant further study (Table 1).

Selection of stimulation parameters form a critical part of any successful DBS application. Besides the SANTE trial, only small cohort studies have reported data on stimulation settings. Usually, starting frequencies were set between 90 and 185 Hz, with a pulse width between 60 and 150 μs and amplitudes between 1 and 10 V; most studies utilized alternate cycling. Importantly, most studies described adjustments made at the “physicians discretion” [61]. Clearly, further trials with larger cohorts are required to formulate guidelines on optimal programming.

As in other applications of DBS, both target selection and precision of electrode placement are crucial steps for treatment success. Ongoing development and refining of MRI acquisition protocols now allow for an accurate ANT visualization [62], which was unavailable at the time of SANTE, thus raising questions on exact electrode positioning [63], a procedure that may be particularly challenging in TLE, as ANT together with medial dorsal, and medial pulvinar nuclei may undergo atrophy [64] with a subsequent decrease in thalamo-hippocampal connectivity [65]. Despite rather extensive clinical use, DBS mechanisms of action remain poorly characterized, particularly in the context of drug-refractory epilepsy. It seems plausible that both disrupting or artificially driving activity within given critical network hubs could re-establish functional integrity of said circuit, leading to an observable clinical benefit [53].

Yu and co-workers recently demonstrated desynchronization of large-scale epileptic networks following high-frequency ANT stimulation. Importantly, stimulation was found to suppress pathological HC seizure activity and disrupted connectivity across various cortical areas, leading to an overall reduction in seizure frequency [66]. Previous reports have proposed modulating effects of DBS on hippocampal activity, leading to a reduction in network excitability and suppression of seizure activity [67]. Indeed, modulation of functional connectivity within cortical networks has been demonstrated in DBS for movement disorders as well [68]. Therefore, individual connectome analysis could be particularly useful for neuroimaging-informed DBS planning in non-resective, drug-refractory epilepsy.

High-quality connectome datasets have already been harnessed to identify connectivity patterns associated with favorable treatment response in movement disorders [69]. More recently, resting-state fMRI-derived functional connectivity patterns in patients responding to ANT-DBS were found to exhibit strong correlations with the default mode network and anticorrelations with the hippocampus compared to non-responders, potentially due to a DBS-induced increase in seizure propagation thresholds within larger-scale networks [70]. Furthermore, TLE patients can be successfully lateralized according to individual thalamocortical connectivity profiles, pointing towards an importance of thalamocortical networks for seizure spread [71]. More recently, building on results of SANTE, Schaper and co-workers demonstrated improved seizure control by targeting the ANT-mamillothalamic junction, thus effectively modulating a white matter tract [72]. Target identification and trajectory planning may benefit from tractography-informed “cable modeling” [73], i.e., subtracting ictal from inter-ictal diffusion-tensor imaging to allow for an individualized seizure network modulation [74]. Apart from its potential to activate larger neuron populations through axonal stimulation, white matter tract stimulation generally requires lower currents, potentially reducing side effects [75].

Elucidation of specific hub-to-hub and network interactions harbors potential to significantly improve therapeutic response [76]. Interestingly, a strong relationship between the absence of a structural brain lesion on MRI and DBS treatment response has been observed for both ANT- and HC-DBS [8], highlighting the importance of a network-centered target selection. Reflecting this ongoing trend, comprehensive imaging processing pipelines are now available to facilitate connectome analysis for DBS [77].

Finally, it should be noted that, despite its success and growing clinical use, DBS may not be universally offered to all patients with refractory seizures who are ineligible for resective surgery. Although evidence on ideal candidate selection is still very limited, contraindications to ANT-DBS therapy in epilepsy generally include progressive disease etiologies, coexisting psychiatric disorders, frequent psychogenic seizures, MRI and surgical contraindications as well as poor patient compliance. It is thus critical to conduct interdisciplinary case discussions to weigh the expected benefits against potential risks of ANT-DBS treatment in order to define realistic therapy goals [61].

### 3.2. Responsive Neurostimulation

Responsive neurostimulation (RNS) constitutes another form of implantable electrical current delivery. Contrasting the open-loop concept of DBS, RNS (Neuropace Inc., Mountain View, CA, USA) is event-triggered and delivers stimulation after detection of predefined seizure biomarkers based on electrocorticography (closed-loop) [78]. The device continuously monitors EEG data and uses a variety of features, e.g., line length, band-pass filters and area under the curve to detect epileptiform activity. Both depth electrodes and cortical strips are utilized to deliver stimulation to their respective target following recognition of a triggering event. In a 2011 randomized controlled trial, a significant frequency reduction of disabling partial seizures as well as an improved quality of life was observed in patients with drug-refractory partial seizures with a maximum of 2 independent epileptogenic foci [79]. Moreover, favorable long-term outcomes were equally observed at follow-ups [49]. Notably, almost half of the study cohort consisted of patients with mesiotemporal seizure onsets. Given these encouraging results, the US Food and Drug Administration recently approved the RNS system for clinical use in refractory partial onset seizures [80]. Its tremendous initial success and non-destructive nature make RNS particularly well suited for seizures within eloquent areas [9]. Apart from its obvious role as an alternative treatment option in non-resective epilepsy, RNS offers the added benefit of providing long-term, ambulatory EEG recordings for up to 8 years, after which the stimulator battery needs to be replaced. Recently, Hirsch and co-workers harnessed this data source to find the leading temporal lobe in patients with bilateral TLE, which resulted in a successful resection in 17 patients (Table 1). Most remained seizure-free after surgery without the need for RNS, whereas a subgroup was seizure-free with continuing use of it [81]. While EEG data obtained from the RNS system could provide additional information on TLE seizure patterns which might have been missed during routine video-EEG monitoring, it seems far better to push the limits of imaging methods to lateralize the focus instead of using an invasive procedure. Some evidence points towards improved cognitive functioning following RNS therapy, such as verbal memory with neocortical seizures and improved overall memory performance in patients with TLE [82]. Additionally, results from a recently published long-term observational study confirmed sustained reduction in seizure frequencies and improved quality of life [83].

Despite its success, several challenges remain for routine RNS utilization, which need to be addressed in future studies. Adequate target localization and definition of the stimulation zone is crucial for successful RNS therapy. Many centers use bilateral depth electrodes for these purpose, an invasive and risky procedure which offers limited sampling [84]. Moreover, observational studies reported RNS as being most effective in patients with MRI-visible lesions mostly in eloquent areas [9], while cortical surfaces which can be stimulated are far smaller than any resection, potentially leading to reduced effectiveness. Importantly, it is currently unknown whether stimulation needs to be delivered directly at the seizure focus, or to relevant propagation pathways or networks [9]. Consequently, several groups are now attempting to deliver stimulation directly to a critical node within the seizure-generating network [50]. Naturally, RNS would benefit greatly from an extensive “sensor network” to continuously monitor and modulate multiple nodes and hubs to fully understand the seizure dynamics. The exact mode of action for RNS remains yet to be ascertained and is most likely multifactorial. GABA-mediated hyperpolarization or neuronal depolarization blockade by accumulation of extracellular potassium ions might account for stimulation effects on seizure generation and propagation [85]. Additionally, an observed decrease in seizure frequency over time suggests that stimulation might alter gene expression patterns or modulate brain network architecture and connectivity, an appealing hypothesis that needs to be tested [86].

### 3.3. Vagus Nerve Stimulation

Vagus nerve stimulation (VNS) differs in many ways to the two highly invasive methods outlined above. First and foremost, VNS is targeted towards the peripheral part of a cranial nerve, thus carrying fewer risks than any other treatment involving a craniotomy. With >100.000 implanted devices, VNS has emerged as an effective, safe and well-tolerated intervention for certain patients with intractable seizures, particularly children with partial or generalized epilepsy [87]. However, inability to predict individual response and variability in seizure control rates could potentially expose candidates to aesthetic and surgical risks with an uncertain success rate. Therefore, imaging-derived biomarkers are needed to identify suitable candidates for VNS treatment.

Significant progress has been made in recent years in the understanding of the neurobiology underlying VNS response. While initial attempts mostly relied on protein detection and enzyme tracing methods, multimodal neuroimaging now allows for an increasingly detailed characterization of the “vagus afferent network” (VAN), leading to the identification of critical brainstem nuclei and circuitry [88]. Given its peripheral nature, VNS is particularly well suited to study the impact of neurostimulation on brain networks, as high-field MRI can be safely performed after implantation.

By combining connectomic profiling with machine learning, Mithani and co-workers were able to establish predictive factors for VNS treatment response. The authors harnessed both functional and structural connectivity data in combination with support-vector-based machine to accurately predict treatment response (Table 1). Efficacy of VNS was found to be associated with preserved white matter microstructure in several left-lateralized tracts of the VAN, which more closely resemble those of healthy subjects. Moreover, functional connectivity analysis revealed a left insular/temporal network associated with favorable treatment response [89]. Notably, clinical parameters alone were found to be insufficient in predicting treatment response.

Enhanced connectivity between the thalamus, anterior cingulate and insular cortices has been associated with better response to VNS [90]. Furthermore, a shorter duration of epilepsy seems to correlate with treatment response, which could indicate that the seizure network is more easily modifiable close to disease onset. Clearly, neuroimaging, and in particular, connectomics, have the potential to further improve our understanding of the longitudinal structural circuitry changes induced by VNS, thus improving patient selection and candidate counseling (Table 1).

**Table 1:** Landmark papers in neuromodulatory treatment for refractory epilepsy.

Landmark Publications in Neuromodulatory Treatment for Refractory Epilepsy		Key Findings
**Deep Brain Stimulation**		
Fisher et al. [57]	SANTE trial, Epilepsia 2010	ANT-DBS is effectice in reducing seizure frequencies in drug-refractory patients without the option of resective surgery
Salanova et al. [59]	Epilepsia 2015	Up to 76% total decrease in seizure frequency has been demonstrated in long-term follow-up of SANTE patients
Tröster et al. [60]	Seizure 2017	ANT-DBS and associated reduction in seizure frequency improves executive functioning, memory, attention and mood
Yu et al. [66]	Brain 2018	ANT-DBS desynchronization of seizure networks is associated with reduction of seizure frequency, supresses pathological HC activity
Middlebrooks et al. [70]	Neurosurgical Focus 2018	Functional imaging-derived connectivity profiles predict treatment response to ANT-DBS
Schaper et al. [72]	Neurosurgery 2020	Delivering DBS to the mamillothalamic tract junction instead of the ANT surpresses seizure activity, potential target site
**Responsive Neurostimulation**		
Morrell et al. [79]	RNS trial, Neurology 2011	Decrease in disabling partial seizures, improved quality of life in drug-refractory patients with ≤ 2 independent epileptogenic foci
Bergey et al. [49]	Neurology 2015	Long-term efficacy and safety in RNS trial patients
Hirsch et al. [81]	Epilepsia 2020	Long-term ambulatory EEG-sampling obtained from RNS leads provides additional information to lateralize seizures
Loring et al. [82]	Epilepsia 2015	Improved cognitive functioning observed in several domains, i.e., verbal memory; overall memory
Nair et al. [83]	Neurology 2019	Long-term improvement in quality of life and sustained reduction in seizure frequency, 9-year follow-up
**Vagus Nerve Stimulation**		
Morris et al. [87]	AAN guidelines/Neurology 2013	Effective and safe in patients with intractable partial or generalized seizures, ≥ 50% sustained seizure frequency reduction
Hachem et al. [88]	Neurosurgical Focus 2018	Identification of the vagus-afferent network and associated brain stem nuclei
Mithani et al. [89]	Annals of Neurology 2018	Connectivity profiles of insular and temporal networks and preserved white matter microstructure predict treatment response to VNS
Ibrahim et al. [90]	Neuroimage Clinical 2017	Enhanced connectivity between thalamus, anterior cingulate, and insular cortices is associated with favorable VNS response

SANTE—Stimulation of the Anterior Nucleus of the Thalamus in Epilepsy; ANT—Anterior Nucleus of Thalamus; DBS—Deep Brain Stimulation; HC—hippocampus; EEG—electroencephalography; RNS—Responsive Neurostimulation; VNS—Vagus Nerve Stimulation; AAN—American Academy of Neurology.

## 4. Conclusions

Neurostimulation has emerged as an efficacious, safe and well-tolerated treatment modality, thus greatly enhancing the neurosurgeon’s armamentarium, especially for patients where curative surgery is not possible or might have failed. While significant advances have been made in neurostimulation, no single modality has yet reached the effectiveness of resective surgery. Notwithstanding its proven clinical effectiveness, neurostimulation still faces significant challenges. Most importantly, candidate selection for ANT-DBS and RNS has not been standardized as of yet and largely depends on institutional procedures; indeed, robust guidelines currently only exist for VNS. Future research should therefore be directed at advancing our understanding of epileptogenic networks, facilitating development of robust biomarkers to allow for defined selection criteria and formulation of guidelines. Network-informed neurostimulation and individualized connectomics nevertheless harbor the potential to greatly improve the seizure outcome in difficult-to-treat epilepsies.
